# Migraines Are Correlated with Higher Levels of Nitrate-, Nitrite-, and Nitric Oxide-Reducing Oral Microbes in the American Gut Project Cohort

**DOI:** 10.1128/mSystems.00105-16

**Published:** 2016-10-18

**Authors:** Antonio Gonzalez, Embriette Hyde, Naseer Sangwan, Jack A. Gilbert, Erik Viirre, Rob Knight

**Affiliations:** a Department of Pediatrics, University of California San Diego, San Diego, California, USA; b Department of Surgery, University of Chicago, Chicago, Illinois, USA; c Department of Neurosciences, University of California San Diego, San Diego, California, USA; d Department of Computer Science and Engineering, University of California San Diego, San Diego, California, USA; UC Davis Genome Center

**Keywords:** headaches, microbiome, migraines, nitrate reductases

## Abstract

Nitrates, such as cardiac therapeutics and food additives, are common headache triggers, with nitric oxide playing an important role. Facultative anaerobic bacteria in the oral cavity may contribute migraine-triggering levels of nitric oxide through the salivary nitrate-nitrite-nitric oxide pathway. Using high-throughput sequencing technologies, we detected observable and significantly higher abundances of nitrate, nitrite, and nitric oxide reductase genes in migraineurs versus nonmigraineurs in samples collected from the oral cavity and a slight but significant difference in fecal samples.

**IMPORTANCE** Recent work has demonstrated a potentially symbiotic relationship between oral commensal bacteria and humans through the salivary nitrate-nitrite-nitric oxide pathway (C. Duncan et al., Nat Med 1:546–551, 1995, http://dx.doi.org/10.1038/nm0695-546). Oral nitrate-reducing bacteria contribute physiologically relevant levels of nitrite and nitric oxide to the human host that may have positive downstream effects on cardiovascular health (V. Kapil et al., Free Radic Biol Med 55:93–100, 2013, http://dx.doi.org/10.1016/j.freeradbiomed.2012.11.013). In the work presented here, we used 16S rRNA Illumina sequencing to determine whether a connection exists between oral nitrate-reducing bacteria, nitrates for cardiovascular disease, and migraines, which are a common side effect of nitrate medications (U. Thadani and T. Rodgers, Expert Opin Drug Saf 5:667–674, 2006, http://dx.doi.org/10.1517/14740338.5.5.667).

## Abstract (precis)

Recent work has demonstrated a potentially symbiotic relationship between oral commensal bacteria and humans through the salivary nitrate-nitrite-nitric oxide pathway (C. Duncan et al., Nat Med 1:546–551, 1995, http://dx.doi.org/10.1038/nm0695-546). Oral nitrate-reducing bacteria contribute physiologically relevant levels of nitrite and nitric oxide to the human host that may have positive downstream effects on cardiovascular health (V. Kapil et al., Free Radic Biol Med 55:93–100, 2013, http://dx.doi.org/10.1016/j.freeradbiomed.2012.11.013). In the work presented here, we used 16S rRNA Illumina sequencing to determine whether a connection exists between oral nitrate-reducing bacteria, nitrates for cardiovascular disease, and migraines, which are a common side effect of nitrate medications (U. Thadani and T. Rodgers, Expert Opin Drug Saf 5:667–674, 2006, http://dx.doi.org/10.1517/14740338.5.5.667).

## Observation

### Nitrate associations with headaches and migraines.

Nitrate-containing compounds have been identified as common headache triggers. Food preservatives are frequently identified triggers for those who suffer from migraines (1). Also, cardiac medications containing nitrates may cause severe headaches, which occur in over 80% of patients taking them. Indeed, approximately 10% of patients cannot tolerate nitrate therapies due to unbearable headaches (2). Nitrate-induced headaches typically manifest in one of two ways: “immediate” headaches with mild to medium severity developing within an hour of medication ingestion and “delayed” headaches occurring 3 to 6 h after nitrate intake that are much more severe, with migrainelike symptoms (3, 4). Delayed migraines appear to be dose dependent and are more likely to occur in individuals with a family history of migraines (5). The primary literature suggests two differing mechanisms behind these two headache types. Immediate headaches appear to be connected to nitric oxide (NO)-mediated vasodilation; in contrast, delayed migraines, similarly to migraines triggered by foods, stress, and other factors, appear to be activated by the release of calcitonin gene-related peptide (CGRP), glutamate, cyclic GMP (cGMP), or S-nitrosylation-mediated changes in ion channel function (5). Notably, S-nitrosylation is dependent on the presence of NO.

### Nitrate-reducing bacteria in the oral and fecal samples of the AGP.

Because only bacteria, and not human cells, can reduce nitrate to nitrite (6), this may represent a symbiotic relationship by which our oral microbes maintain cardiovascular health using molecules present in our food. It has also been reported that in murine macrophages *in vitro*, the bacterial nitric oxide reductase NorB increases the decomposition rate of *S*-nitrosothiol (SNO) (7). This represents a potential connection between nitric oxide reductases and nitrate-induced migraines. Therefore, we determined the presence and abundance of nitrate, nitrite, and nitric oxide reductase genes in predicted metagenomes from stool and oral samples in the American Gut Project (AGP) cohort and correlated these genes with self-reported migraine status.

Using a subset of 16S rRNA data from sequencing rounds 1 to 25 of the public American Gut Repository (ftp://ftp.microbio.me/AmericanGut/rounds-1-25; subset details are described in Text S1 in the supplemental material), we used analysis of composition of microbiomes (ANCOM) (8) to identify GreenGenes (GG, 97% similarity) operational taxonomic units (OTUs) that were differentially abundant between migraineurs and nonmigraineurs. We next predicted the metagenome functional content of the entire group of differentially abundant OTUs using Phylogenetic Investigation of Communities by Reconstruction of Unobserved States (PICRUSt) (9). To assess the accuracy of the predictions, we calculated the nearest sequenced taxon index (NSTI), which calculates the average branch length separating each OTU in a sample from a reference bacterial genome, weighted by the abundance of that OTU in the sample. The NSTI scores were good (below 0.10) for both stool and oral samples, indicating appropriate metagenome predictions, which were stronger in oral samples than in stool samples (see Fig. S1 in the supplemental material).

10.1128/mSystems.00105-16.1 Figure S1  Nearest sequenced taxon index (NSTI). Fecal and oral NSTI per migraine status, which shows predictions of gene composition range from quite good to good. Download Figure S1, EPS file, 1.8 MB. Copyright © 2016 Gonzalez et al. 2016 Gonzalez et al. This content is distributed under the terms of the Creative Commons Attribution 4.0 International license.

10.1128/mSystems.00105-16.2 Text S1  Detailed analytical steps for all analyses. Additionally, all commands and analyses performed in the manuscript can be found at: https://github.com/knightlab-analyses/mSystems00105-16. Download Text S1, DOCX file, 0.1 MB. Copyright © 2016 Gonzalez et al. 2016 Gonzalez et al. This content is distributed under the terms of the Creative Commons Attribution 4.0 International license.

**FIG 1:**
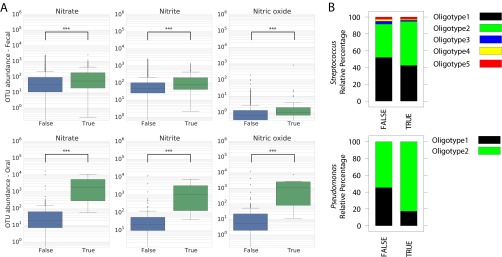
Nitrate-, nitrite-, and nitric oxide-reducing bacteria. (A) Differential abundances of OTUs as detected by ANCOM that have nitrate-, nitrite-, and nitric acid-producing KEGG orthologies (KOs) as reported by PICRUSt by body site. Oral samples show obvious differences between migraineurs (True) and nonmigraineurs (False) in nitrate, nitrite, or NO reductase genes, while there are no obvious differences in stool samples. However, both nitrate and nitrite are significant in stool samples, but not nitric oxide, as sample groups are too small. (B) Relative abundance profiles of oligotypes (sub-OTUs) in *Streptococcus* (two oligotypes) and *Pseudomonas* (five oligotypes). Intergroup analysis revealed no major differences in the *Streptococcus* distribution profiles. *Pseudomonas* oligotype 2 was highly enriched in the “TRUE” group (FALSE = 54%, TRUE = 82%).

Given the role of the oral microbiome in nitrate reduction and the association between nitrates and headaches, we hypothesized that the abundances of nitrate, nitrite, and NO reductase genes in the predicted metagenomes in oral and stool samples would differ significantly between migraineurs and nonmigraineurs. As seen by the results in Fig. 1A, there were small but significant increases (Kruskal-Wallis: nitrate, *P* ≤ 0.001; nitrate, *P* ≤ 0.001; and nitric oxide, *P* ≤ 0.001) in nitrate, nitrite, and nitric oxide reductase genes in stool samples collected from migraineurs, and in oral samples, nitrate, nitrite, and nitric oxide reductase genes were all significantly (Kruskal-Wallis: nitrate, *P* ≤ 0.001; nitrite, *P* ≤ 0.001; and nitric oxide, *P* ≤ 0.001) more abundant (based on ANCOM) in migraineurs.

The dominant oral OTUs (>10% of the reads in the data set) that were significantly different between migraineurs and nonmigraineurs belonged to the genera *Streptococcus* and *Pseudomonas*, both of which have species with the potential to reduce nitrate (10, 11). Additionally, while *Pseudomonas* has not previously been reported in the context of oral nitrate reduction, *Streptococcus* did increase in the oral cavities of rats supplemented with nitrate in their drinking water (12). To explore whether there were strain-level differences within these genera across the populations, we performed oligotyping (13). The genera *Streptococcus* and *Pseudomonas* were decomposed into 5 and 2 oligotypes, respectively (Fig. 1B). There was no significant difference in the relative abundance patterns of *Streptococcus* oligotypes across both populations. Two-group analysis (Fisher’s exact *t* test, *P* < 0.005) suggested that *Pseudomonas* (10% of the total reads) has differential abundance patterns in the oral microbiome of migraineurs and nonmigraineurs. *Pseudomonas* oligotypes 1 and 2 were both present in both populations, but oligotype 2 was significantly more abundant in migraineurs. These results indicate that host preference patterns in the genus *Pseudomonas* are driven by host physiology and that migraineurs share similar strains of *Pseudomonas*.

Finally, based on the extrapolation of the strain level profiles of *Pseudomonas* oligotypes and the genomic variations found via PICRUSt, it is likely that these strains comprise a genetic repertoire selected for genetic adaptation in this host environment (migraine).

We next determined the taxonomic classification of OTUs contributing nitrate, nitrite, and nitric oxide reductase genes to the predicted metagenomes in our datasets. From this list, two bacterial taxa (*Rothia mucilaginosa* and *Haemophilus parainfluenzae*) have previously been reported as some of the main nitrate reducers in the human oral cavity (12, 14), and some have also been reported to be associated with headaches (Table 1).

**TABLE 1:** GreenGenes identification numbers and taxonomy assignments of the five most common OTUs that were found to be differentially abundant between migraineur status and whether they contain nitrate-, nitrite-, and/or nitric oxide-reducing KEGG orthologies

GG ID^a^	GG taxonomy	OTU contributes:			Reference^b^
		Nitrate reductase(s)	Nitrite reductase(s)	Nitric oxide reductase(s)	
903426	k__Bacteria; p__Actinobacteria; c__Actinobacteria; o__Actinomycetales; f__Micrococcaceae; g__Rothia; s__mucilaginosa	X	X	X	15
926526	k__Bacteria; p__Actinobacteria; c__Actinobacteria; o__Actinomycetales; f__Micrococcaceae; g__Rothia; s__mucilaginosa	X	X	X	15
538000	k__Bacteria; p__Proteobacteria; c__Gammaproteobacteria; o__Enterobacteriales; f__Enterobacteriaceae; g__; s__	X	X		
960871	k__Bacteria; p__Actinobacteria; c__Actinobacteria; o__Actinomycetales; f__Actinomycetaceae; g__Actinomyces; s__	X			
4448331	k__Bacteria; p__Proteobacteria; c__Gammaproteobacteria; o__Enterobacteriales; f__Enterobacteriaceae; g__; s__	X	X	X	
4299925	k__Bacteria; p__Proteobacteria; c__Gammaproteobacteria; o__Pasteurellales; f__Pasteurellaceae; g__Haemophilus; s__parainfluenzae		X		16
821562	k__Bacteria; p__Proteobacteria; c__Gammaproteobacteria; o__Pseudomonadales; f__Pseudomonadaceae; g__; s__			X	
4128270	k__Bacteria; p__Proteobacteria; c__Gammaproteobacteria; o__Pseudomonadales; f__Pseudomonadaceae; g__Pseudomonas; s__			X	

^a^ GG, GreenGenes; ID, identification number.

^b^ For those taxonomies with a species name, we provide references to reports of their relationship with headache studies.

### Conclusions.

These results show for the first time a potential link between bacterial nitrate, nitrite, and nitric oxide reducers and migraines, by reporting their higher abundances in the oral cavities of people with migraines than in the oral cavities of those who do not suffer from migraines. Future studies should focus on further characterizing the connection between oral bacterial nitrate, nitrite, and nitric oxide reducers and migraines.
