# Correction for Gonzalez et al., “Migraines Are Correlated with Higher Levels of Nitrate-, Nitrite-, and Nitric Oxide-Reducing Oral Microbes in the American Gut Project Cohort”

**DOI:** 10.1128/mSystems.00023-17

**Published:** 2017-04-11

**Authors:** Antonio Gonzalez, Embriette Hyde, Naseer Sangwan, Jack A. Gilbert, Erik Viirre, Rob Knight

**Affiliations:** a Department of Pediatrics, University of California San Diego, San Diego, California, USA; b Department of Surgery, University of Chicago, Chicago, Illinois, USA; c Department of Neurosciences, University of California San Diego, San Diego, California, USA; d Department of Computer Science and Engineering, University of California San Diego, San Diego, California, USA

## AUTHOR CORRECTION

Volume 1, no. 5, e00105-16, 2016, https://doi.org/10.1128/mSystems.00105-16. We have been following some of the comments about this paper and accept that the wording of parts of our paper might be interpreted in ways that we did not intend and that do not reflect the work performed. We want to make it clear that for this paper, we made predictions about nitrate-, nitrite-, and nitric oxide-reducing oral microbes in the American Gut Project Cohort based on analysis of rRNA amplicon sequences and matching them to known genomes. We did not directly measure these genes involved in nitrate metabolism (nitrate reductase, nitrite reductase, and nitric oxide reductase) or know for certain that the strains present in the samples have such functions (although they are widely distributed in the matching phylogenetic groups). Some of the wording (e.g., of the title and the abstract) did not come across as we intended and could be interpreted as implying that we made direct measurements. We believe that the predictions that we made are useful but acknowledge that they have limitations. We also want to stress that to test these hypotheses and advance clinical practice, we would need to extensively validate our results through intervention studies of carefully controlled clinical populations, which is obviously considered beyond the scope of the Observation format. However, we are currently performing studies that we believe will advance this research, including some work based on public comments made about the lack of validation of the specific claims of the paper.

