# Supplementary material for: Migraines Are Correlated with Higher Levels of Nitrate-, Nitrite-, and Nitric Oxide-Reducing Oral Microbes in the American Gut Project Cohort
Source: mSystems. 2016 Oct 18;1(5):e00105-16. doi: 10.1128/mSystems.00105-16 (PMC5080405; doi:10.1128/mSystems.00105-16)
Supplement: Text S1 [file sys005162059s2.docx]

**Supplementary material**

***Description of cohort***

We accessed sequences and metadata from sequencing rounds 1 to 25 of the public American Gut repository ([ftp.microbio.me/AmericanGut/rounds-1-25/](ftp://ftp.microbio.me/AmericanGut/rounds-1-25/)). Following the method described by the online analytical methods on the American Gut cohort, we filtered the cohort to conserve samples based on the following criteria: adults 20-69 years of age with BMIs ranging between 18.5 and 30, and no self-reported history of inflammatory bowel disease, diabetes, or antibiotic use in the past year. Samples that were mislabeled based on the Human Microbiome Project protocol (1), and any sample with under 10,000 sequences were also discarded. The resulting OTU table was then split by sample type and all analyses were performed on stool and oral samples separately. We then split the cohort into two groups based on migraine status. Those participants who self reported a diagnosis of migraines by a medical professional, alternative medicine practitioner, or self-diagnosed were included in the migraine “TRUE” group (stool, n=171; oral, n=6); those that did not self-report a migraine diagnosis (no diagnosis or question left unanswered) were included in the migraine “FALSE” group (stool, n=1825; oral, n=166).

***ANCOM***

We used the analysis of composition of microbes (ANCOM) (2) to identify differentially abundant OTUs across migraine groups. ANCOM uses the samples intrinsic structure to compare the composition of microbes in the population. The next analytical steps were performed on the subset of OTUs that were marked as differentially abundant between samples.

***PICRUSt metagenome predictions***

The metagenome functional content was predicted from the 16S rRNA sequences using PICRUSt (3) and the OTU abundance of nitrate-nitrite-nitric oxide reducing bacteria was plotted. These plots and their significance was tested using the Kruskal-Wallis test as implemented SciPy (4). To assess prediction accuracy, we calculated the Nearest Sequenced Taxon Index, which calculates the average branch length separating each OTU in a sample from a reference bacterial genome, weighted by the abundance of that OTU in the sample. As described by (5), NSTI scores under 0.06 are quite good, while scores between 0.06-0.10 are good, and scores above 0.20 indicate inaccurate predictions.

***Oligotyping***

We used the oligotyping pipeline (6) to identify the sub-OTU-level differences across the genera *Streptococcus* and *Pseudomonas* i.e., the two most differentially abundant genera across oral samples (*Pseudomonas*=23.6%, *Streptococcus*=11.1%). A two-sided Fisher’s exact test was used to compare the abundance patterns of oligotypes assembled across migraineurs and non-migraineurs.

**References**

1. **Human Microbiome Project C.** 2012. Structure, function and diversity of the healthy human microbiome. Nature **486:**207-214.

2. **Mandal S, Van Treuren W, White RA, Eggesbo M, Knight R, Peddada SD.** 2015. Analysis of composition of microbiomes: a novel method for studying microbial composition. Microb Ecol Health Dis **26:**27663.

3. **Langille MG, Zaneveld J, Caporaso JG, McDonald D, Knights D, Reyes JA, Clemente JC, Burkepile DE, Vega Thurber RL, Knight R, Beiko RG, Huttenhower C.** 2013. Predictive functional profiling of microbial communities using 16S rRNA marker gene sequences. Nat Biotechnol **31:**814-821.

4. **Jone E, Oliphant T, Peterson P, others.** 2001. SciPy: Open Source scientific tools for Python.

5. **Zaneveld JR, Lozupone C, Gordon JI, Knight R.** 2010. Ribosomal RNA diversity predicts genome diversity in gut bacteria and their relatives. Nucleic Acids Res **38:**3869-3879.

6. **Eren AM, Maignien L, Sul WJ, Murphy LG, Grim SL, Morrison HG, Sogin ML.** 2013. Oligotyping: Differentiating between closely related microbial taxa using 16S rRNA gene data. Methods Ecol Evol **4**.
